# Metabarcoding Is Powerful yet Still Blind: A Comparative Analysis of Morphological and Molecular Surveys of Seagrass Communities

**DOI:** 10.1371/journal.pone.0117562

**Published:** 2015-02-10

**Authors:** Dominique A. Cowart, Miguel Pinheiro, Olivier Mouchel, Marion Maguer, Jacques Grall, Jacques Miné, Sophie Arnaud-Haond

**Affiliations:** 1 IFREMER (Institut Français de Recherche pour l’Exploitation de la MER), Unité Environnement Profond, Département des Ressources physiques et Ecosystèmes de Fond de mer (REM), B.P. 70, 29280, Plouzané, France; 2 University of St. Andrews, Medical and Biological Sciences Building, North Haugh, St. Andrews, Fife, KY16 9TF, United Kingdom; 3 Institut Universitaire Européen de la Mer (IUEM), Technopôle Brest-Iroiserue Dumont d’Urville, 29280, Plouzané, France; 4 Total Exploration & Production, Direction HSE, 2 Place Jean Millier, 92078, Paris la Défense, France; Università della Calabria, ITALY

## Abstract

In the context of the sixth wave of extinction, reliable surveys of biodiversity are increasingly needed to infer the cause and consequences of species and community declines, identify early warning indicators of tipping points, and provide reliable impact assessments before engaging in activities with potential environmental hazards. DNA metabarcoding has emerged as having potential to provide speedy assessment of community structure from environmental samples. Here we tested the reliability of metabarcoding by comparing morphological and molecular inventories of invertebrate communities associated with seagrasses through estimates of alpha and beta diversity, as well as the identification of the most abundant taxa. Sediment samples were collected from six *Zostera marina* seagrass meadows across Brittany, France. Metabarcoding surveys were performed using both mitochondrial (Cytochrome Oxidase I) and nuclear (small subunit 18S ribosomal RNA) markers, and compared to morphological inventories compiled by a long-term benthic monitoring network. A sampling strategy was defined to enhance performance and accuracy of results by preventing the dominance of larger animals, boosting statistical support through replicates, and using two genes to compensate for taxonomic biases. Molecular barcodes proved powerful by revealing a remarkable level of diversity that vastly exceeded the morphological survey, while both surveys identified congruent differentiation of the meadows. However, despite the addition of individual barcodes of common species into taxonomic reference databases, the retrieval of only 36% of these species suggest that the remaining were either not present in the molecular samples or not detected by the molecular screening. This finding exemplifies the necessity of comprehensive and well-curated taxonomic reference libraries and multi-gene surveys. Overall, results offer methodological guidelines and support for metabarcoding as a powerful and repeatable method of characterizing communities, while also presenting suggestions for improvement, including implementation of pilot studies prior to performing full “blind” metabarcoding assessments to optimize sampling and amplification protocols.

## Introduction

The sixth wave of extinction has already begun, far in advance of the completion of comprehensive biodiversity inventories [1,2]. Awareness of this situation has led to the establishment of transnational conservation programs whose effectiveness rely upon the ability to thoroughly assess biodiversity and provide indicators of ecosystem health within a time frame that counteracts the initial delayed response [3]. The construction of biological inventories has traditionally and primarily relied upon morphological identifications of taxonomic groups, however, morphological discrimination of a given community is a time consuming task that requires meticulous taxonomic expertise that is unfortunately becoming more rare [4,5]. Thus, there is a need for methods that can rapidly and cost effectively appraise ecosystem biodiversity and temporal variations following natural or impacted trajectories [4,6].

The improvement of molecular techniques in recent years have allowed for the development of genetic methods that help increase the rate and accuracy of species identification, at even the most remote ecosystems [7,8]. DNA barcoding, an approach in which target DNA sequences provide accelerated taxonomic identification, discrimination and discovery of unknown organisms, is at the forefront of these investigations [4,9,10]. DNA barcoding has become especially useful for analyses of environmental collections (water, soil, mud, feces, etc…), for which ‘environmental metagenetics’ is implemented when community sorting and morphological descriptions are challenging due to the large number and small sizes of possible taxon, as well as to the state of conserved specimens in samples [11–16].

Identifying early warning indicators of tipping points in ecosystems requires the most comprehensive appraisal of community biodiversity, as species or assemblages able to reveal the approach of critical thresholds may be invisible, cryptic or rare [17–20]. Ecosystem assessments performed by large-scale transnational conservation planning programs such as Natura 2000 (http://www.natura.org/), as well as by private companies, are also increasingly required to develop reliable environmental impact assessments (EIAs) before engaging in new activities. As a result of these requirements, “blind metabarcoding”, or ascribing taxonomic identity directly to sequences, has emerged as a possibly optimal solution for biodiversity surveys and inventories, in terms of costs and time schedules [21,22].

Morphological and molecular approaches have long been considered complementary [23,24], raising legitimate doubts as to the accuracy and reliability of blind metabarcoding. Thus far, several studies have shown the increased time efficiency of molecular techniques [25,26], particularly for specific taxa (fishes: [27], nematods: [28,29], arthropods: [24]). Additionally, several studies have addressed unknown and unrecognizable taxa [7], stomach contents [27,30,31] or microbial diversity [32]. The reliability of “blind metabarcoding” in those studies was tested through the analysis of controlled laboratory admixture or plausibility of taxa uncovered, whereas very few studies have rigorously compared the efficiency of molecular versus *in situ* morphological community descriptions from the same areas sampled for molecular analysis (*see* [24]). Single studies have, however, demonstrated great advances in estimating gains in terms of time saved and biodiversity revealed, by focusing on a single target gene and specific taxonomic groups such as Cytochrome Oxidase I (COI) for birds and arthropods [24], and the small subunit 18S ribosomal RNA region (18S) for nematodes and zooplankton [6,29].

In the present study, we aimed to test the efficiency and reliability of metabarcoding for environmental survey by providing one of the first comprehensive and rigorous comparisons of morphological and molecular characterizations of invertebrate communities. We characterized communities associated with *Zostera marina* seagrass meadows, using both mitochondrial and nuclear ribosomal genetic markers (Cytochrome Oxidase I and the small subunit 18S ribosomal RNA region) to increase the proportion of informative fragments in environmental DNA extracts [33], as well as to compare the diversity estimates between two barcoding genes.

For alpha diversity estimates, the amount of taxonomic units revealed through a single molecular snapshot largely exceeded those uncovered through the morphological survey. Furthermore, the use of multi-gene metabarcoding also provided more reliable estimates of community composition than single genes, as each molecular marker has taxonomic specific affinities. Results also disclosed congruent patterns of beta diversity estimates across molecular and morphological surveys to confirm the reliability of metabarcoding, in addition to the molecular surveys uncovering meiofauna taxa. We also identified pitfalls and suggestions for improvement of molecular inventories, including the need for multi-marker assessments to unravel the broadest possible taxonomic diversity, as well as improved reference databases. Simultaneously, this comprehensive and comparative approach has allowed us to define an optimal sampling design that includes triplicate core samples for testing statistical significance, size trimming to limit the dominance of larger organisms, and a preliminary combination of broad taxonomic spectrum DNA markers to optimize estimates of biodiversity to be further used for ecological applications.

## Methods

### Sediment collections and sample processing

**Fig 1:**
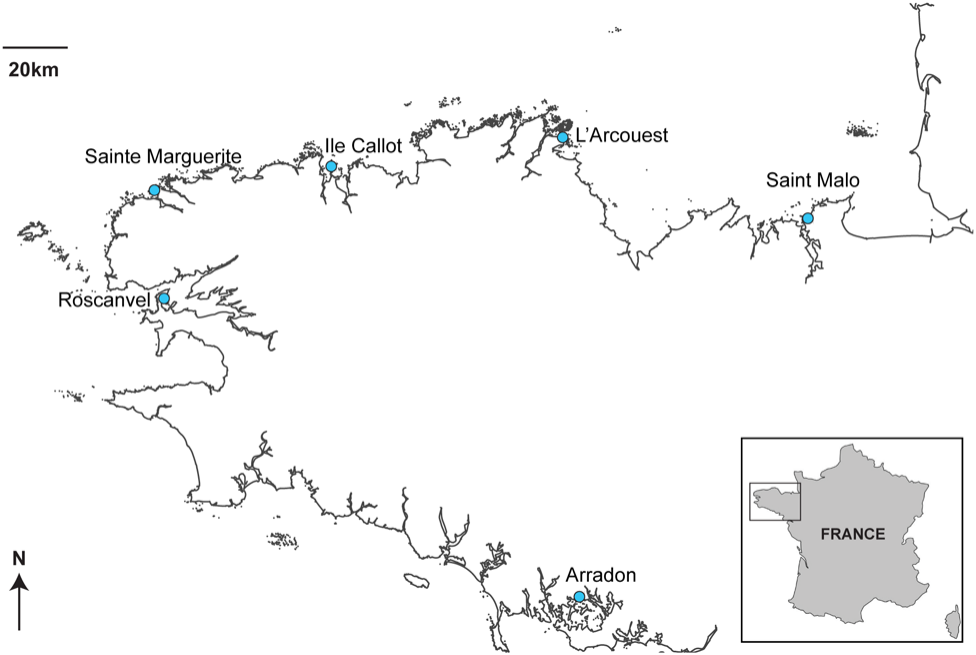
Map of six *Zostera marina* seagrass meadows along the coast of Brittany, France, where sediment collections were performed.

Sediment samples were collected from six *Zostera marina* seagrass meadows along the Brittany coast in western France (Fig. 1). No specific permissions were required to sample sediment at any of these locations, and the fieldwork performed here did not involve endangered or protected species. These meadows have been followed as part of an eight-year benthic survey, in which morphological identifications were performed by the observatory of the Institut Universitaire Européen de la Mer (IUEM), a component of the REseau BENThique (REBENT network [34]). The locations of each meadow are detailed in Table 1. The sampling protocol for the morphological characterization included two main collection methods: I) the collection of mobile epi-macrofauna, which were captured using pushing-nets on 10 m² surface, and II) the collection of soil-dwelling macrofauna, which were collected by sampling of sediments cores (0.03 m²). For the molecular characterization each location, two 20 X 30 m quadrats, that were previously sampled for the morphological survey and spaced several meters apart from one another, were chosen for sampling [35]. Three sediment cores per quadrat were collected during spring high tide at Sainte Marguerite and Arradon in 2010, and Sainte Marguerite, Ile Callot, L’Arcouest, Roscanvel and Saint-Malo in 2011, with the exception of Arradon, where only two cores could be analyzed (Table 1).

**Table 1:** Locations of six *Zostera marina* seagrass meadows from where sediment collections were made. Implemented barcoding markers were Cytochrome Oxidase (COI) and the small subunit 18S ribosomal RNA region (18S).

Meadow	Latitude	Longitude	Year(s)	No. of quadrats	No. of cores	Mesh sizes (mm)	Genes
Saint Malo	48.648° N	2.007° W	2011	2	3	0.5, 1.0, 2.0	COI
L’Arcouest	48.818° N	3.008° W	2011	2	3	0.5, 1.0, 2.0	COI
Ile Callot	48.697° N	3.925° W	2011	2	3	0.5, 1.0, 2.0	COI
Sainte Marguerite	48.596° N	4.623° W	2010/2011	1/2	3/3	0.5, 1.0, 2.0	18S & COI
Roscanvel	48.317° N	4.547° W	2011	2	3	0.5, 1.0, 2.0	COI
Arradon	47.626° N	2.822° W	2010	1	2	0.5, 1.0, 2.0	18S &COI

**Fig 2:**
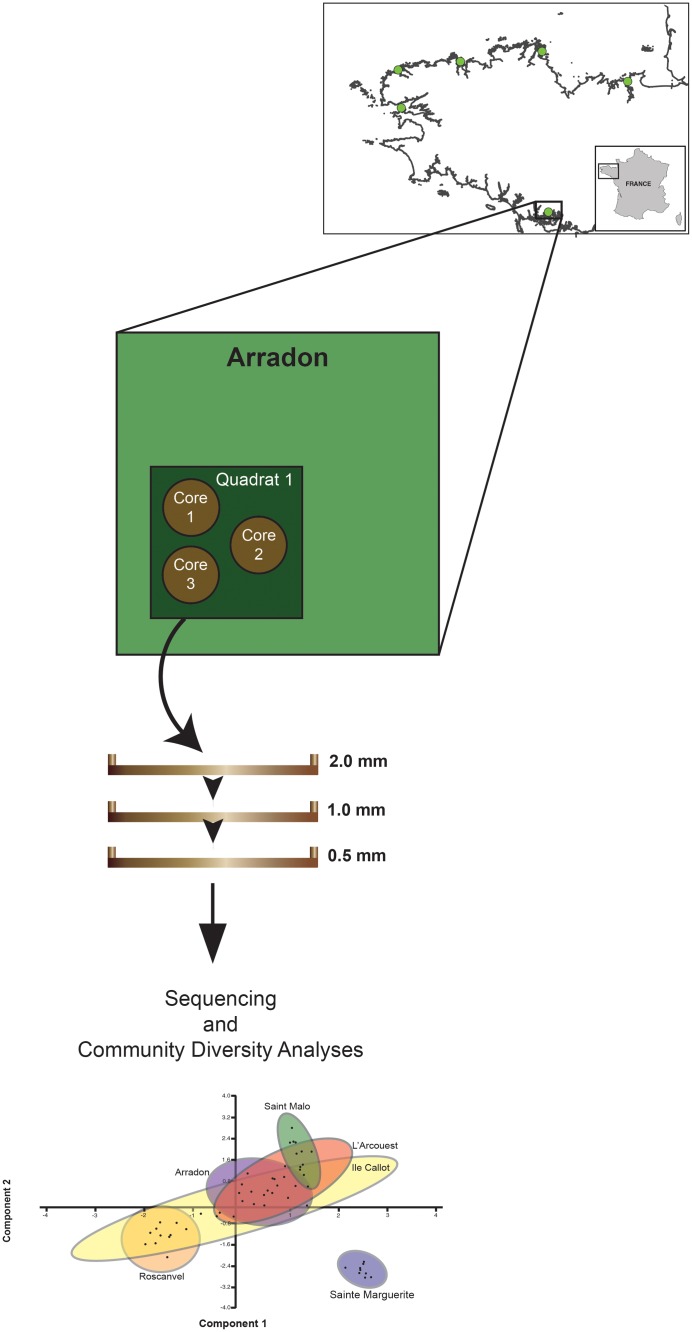
Sediment sampling schematic for the present study.

The exact same quadrates were rigorously chosen and sampled at similar dates for both surveys, yet morphological surveys require net and core sampling of extensive spatial areas, leading to an amount of material (tissues and sediment) not realistic to process for DNA extraction, which requires a smaller amount of homogenized material. To this end, sediment cores measuring 10cm diameter by 15cm depth were sieved on site using local seawater through decreasing mesh sizes of 2mm, 1mm, and 0.5mm in tandem order. This was done to identify if filtering by size uncovers additional metazoan diversity, as well as to prevent the dominance of larger bodied animals (see Fig. 2 for schematic of sampling protocol). As three mesh sizes were analyzed for each core sampled from each meadow, the term ‘sample’ refers to the sediment of a specific mesh size. More specifically, as the sediment remaining in a specific filter was collected as an individual sample, there were three samples collected for each core, two to three cores collected per quadrat, and two quadrats per meadow. Sieved sediment samples were immediately preserved at -80°C in zip-locked bags until DNA extractions were performed on about 10g of each sample (i.e. each sieved fraction of each core), according to the protocol of PowerTM Soil DNA Extraction Kit (MO BIO Laboratory, Solana Beach kits CA, USA). DNA extract quality was assessed using NanoDrop (Thermo Scientific).

Amplifications of two common barcoding genes, Cytochrome Oxidase (COI) and the small subunit 18S ribosomal RNA region (18S), were performed using “universal” primers for COI [36] and 18S [37], obtaining fragments of about 710bp and 450bp, respectively. Degenerate COI primers (F_dgLCO-1490 5’-GGT CAA CAA ATC ATA AAG AYA TYG G and R_dgHCO-2198 5’-TAA ACT TCA GGG TGA CCA AAR AAY CA-3, [38]) were found to be suitable for the universal amplification of the COI fragment by revealing more diversity than the non-degenerated primers in a test of 454 sequencing, and were therefore used to amplify the COI fragment in 105 samples. For 15 of those samples from two sites, Sainte-Marguerite and Arradon (see Table 1), we additionally amplified 18S using the same primers as those previously used for metabarcoding of marine sediments and water samples [6,25,26]. As COI is widely known as the standard barcoding molecule for its ability to discriminate species in many animal groups [9,39] and in addition to Tang and others [5] identifying COI as uncovering more meiofaunal diversity than 18S, we chose to focus on COI for the large scale comparison of morphologically vs. molecularly characterized communities for all six seagrass meadows.

Two PCR replicates were performed for each sample using PolymeraseTwo—Platinum Taq High Fidelity (Invitrogen, CA, USA) from 50 ng of genomic DNA. Amplification was performed using the following PCR conditions: 95°C (2 min), 35 cycles of 95°C (1 min), 57°C (45 s), 68°C (3 min), followed by an elongation step of 68°C for 10 min. PCR products were visualized on 1.5% agarose gels stained with ethidium bromide to confirm the presence of COI and 18S fragments. PCR purification was completed using “Ampure beads” (Beckman Coulter, MA, USA). Amplification products were quantified by fluorimetry using the Invitrogen “PicoGreen” kit (CA, USA), and clustered in equimolar concentrations for sequencing reactions with 454 GS FLX Titanium, as instructed by the manufacturer (Roche, 454 Life Sciences, Branford, CT, USA). Unique sequences of 8bp were used as identifier tags to differentiate pooled samples and sequencing primers during bioinformatics analyses.

### Sequencing of REBENT taxonomically identified metazoans

To determine if commonly observed and morphologically identified species could be uncovered in our molecular datasets, tissues from the 50 most common benthic metazoan species from *Zostera marina* seagrass communities (Table 2) were isolated for DNA extraction and sequencing. The 50 species were collected and identified as a part of the morphological inventory compiled by the IUEM/REBENT network [34], and classified as the most common due to their consistent presence and high abundance at the six seagrass meadows described in this study. A detailed protocol for the extraction and sequencing of these metazoan species can be found in the S1 Protocol in the supplemental material section. A 710bp fragment of COI was successfully amplified and sequenced in 14 of the 50 species, while a 450bp fragment of 18S was successfully amplified and sequenced for 33 of those species, each using the respective universal primer set. As public databases may contain sequence data that has not been taxonomically curated, the isolated sequences we amplified (known as common species “barcodes”) were added to the reference databases used for taxonomic assignments, to increase the accuracy of the assignments.

**Table 2:** The 50 most common invertebrate metazoan found in *Zosteria* seagrass meadow sediment (REBENT, 2010). x denotes taxon found in NCBI GenBank, * denotes taxa barcode added to NCBI GenBank (this study), F (Family), G (Genus) and S (Species) denote taxonomic level found in the COI and 18S environmental sequenced datasets for this study.

Phylum	Class	Family	Species	COI Genera	COI Species	Presence in COI Metadataset	18S Genera	18S Species	Presence in 18S Metadataset
**Annelida**	Polychaeta	Ampharetidae	Melinna palmata	*	*		**x**	*	**S**
		Capitellidae	Notomastus latericeus	**x**			**x**	**x**	**S**
		Capitellidae	Heteromastus filiformis				**x**	**x**	**F**
		Capitellidae	Capitella capitata	**x**	**x**		**x**	**x**	**G**
		Cirratulidae	Caulleriella bioculata				**x**		**F**
		Cirratulidae	Cirriformia tentaculata				**x**	**x**	**F**
		Cirratulidae	Chaetozone setosa	**x**	**x**	**S**			**F**
		Glyceridae	Glycera alba	**x**			**x**	**x**	
		Lumbrineridae	Scoletoma impatiens	**x**					**G**
		Lumbrineridae	Lumbrineris latreilli	**x**	**x**		**x**	**x**	**G**
		Maldanidae	Euclymene oerstedi	**x**	*	**S**	**x**	**x**	**S**
		Maldanidae	Clymenura clypeata	*	*	**S**	**x**	**x**	**S**
		Nephtyidae	Nephtys hombergii	**x**	**x**	**S**	**x**	**x**	**S**
		Nereididae	Platynereis dumerilii	**x**			**x**	**x**	**S**
		Nereididae	Neanthes caudata	**x**			**x**	*	**S**
		Nereididae	Perinereis cultrifera	**x**			**x**	*	**S**
		Orbiniidae	Scoloplos armiger	**x**	**x**		**x**	**x**	**S**
		Phyllodocidae	Phyllodoce mucosa	**x**	**x**	**F**	**x**		**F**
		Phyllodocidae	Eteone longa	**x**	**x**	**F**	**x**	**x**	**G**
		Sabellidae	Megalomma vesiculosum	**x**			**x**	*	**S**
		Sigalionidae	Sthenelais boa	*	*	**S**	**x**	**x**	
		Spionidae	Aonides oxycephala				**x**	**x**	**S**
		Spionidae	Spio martinensis	**x**			**x**		**G**
**Arthropoda**	Malacostracea	Ampeliscidae	Ampelisca brevicornis	**x**	*		**x**	*	
		Ampithoidae	Ampithoe rubricata	**x**	**x**	**S**	**x**	**x**	
		Aoridae	Microdeutopus anomalus					*	
		Apseudidae	Apseudes latreillii	**x**			**x**	*	**S**
		Apseudidae	Apseudes talpa	**x**	**x**		**x**	*	**G**
		Atylidae	Atylus guttatus	*	*		**x**	**x**	
		Atylidae	Atylus swammerdami	*	*		**x**	*	
		Bodotriidae	Iphinoe trispinosa	**x**	**x**		*	*	
		Caprellidae	Phtisica marina				**x**	**x**	
		Caprellidae	Caprella acanthifera	**x**			**x**	*	
		Dexaminidae	Dexamine spinosa	*	*		*	*	
		Hippolytidae	Hippolyte varians				**x**	**x**	
		Lysianassidae	Lysianassa plumosa	**x**		**F**			
		Melitidae	Gammarella fucicola				*	*	
		Paguridae	Anapagurus hyndmanni	*	*		*	*	
		Portunidae	Polybius arcuatus	**x**					
		Tanaidae	Tanais dulongii	**x**	**x**		**x**	*	
		Urothoidae	Urothoe pulchella	*	*		**x**	*	
**Cnidaria**	Anthozoa	Actiniidae	Anemonia viridis		**x**		**x**	**x**	**G**
**Mollusca**	Bivalvia	Lucinidae	Lucinoma borealis				**x**	**x**	**S**
		Montacutidae	Mysella bidentata	*	*		**x**	*	
		Semelidae	Abra alba	**x**			**x**	**x**	
	Gastropoda	Calyptraeidae	Calyptraea chinensis	**x**	**x**	**F**			
		Nassariidae	Nassarius reticulatus	**x**	**x**		**x**	*	
		Trochidae	Gibbula cineraria	**x**	**x**	**F**	**x**	**x**	
		Trochidae	Jujubinus striatus	**x**	**x**	**S**	*	*	
**Sipuncula**	Sipunculidae	Golfingiidae	Golfingia elongata				**x**	**x**	**S**
**Total in NCBI public database**				**39**	**26**	**F(5), G(0), S(5)**	**44**	**41**	**F(5), G(7), S(14)**

### Bioinformatics data processing and analyses

Raw pyrosequenced reads for COI and 18S were processed, clustered and taxonomically assigned, using the Quantitative Insights into Microbial Ecology (QIIME) v. 1.7.0 pipeline [40]. A workflow of QIIME scripts executed for this project can be found in S2 Protocol of the supplemental material section. During the processing stage, dataset demultiplexing and quality checks, including the removal of low quality and short sequences (< 200bp), were implemented using the split_libraries.py script. Filtered reads were clustered into *de novo* molecular operational taxonomic units (MOTUs, [41]) with the aid of UCLUST [42], using the script pick_otus.py in QIIME at a defined pairwise sequence identity cut off value was 97% [5,6,43]. Next, a representative sequence set of the clusters was generated using the pick_rep_set.py script. Chimeras and “quasi-singletons” (sequences appear < 3 times in the dataset) were identified and removed from the MOTU table using USEARCH v.6.1 [42] implemented in QIIME.

The 33 and 14 prescreened and taxonomically confirmed DNA barcode sequences for 18S and COI (accessions KJ182970—KJ183017) were added to previously compiled reference databases for taxonomic assignment. Fourteen (18S) and three (COI) of these prescreened and taxonomically confirmed sequences were already present in the GenBank public database (www.ncbi.nlm.nih.gov/genbank), based only upon their shared sequence names (see Table 2). For 18S, the morphologically confirmed barcodes were added to the SILVA SSU r115 EMBL *eukarya* database ([44], http://www.arb-silva.de/) to produce a reference database composed of 11,617 sequences. The COI barcodes were added to a COI *metazoan*-only reference database that we complied from GenBank to produce a database composed of 48,734 sequences. These databases were used to assign taxonomic identifications to representative MOTU sequences based on the highest similarity score using the BLAST assignment method [45] implemented through the assign_taxonomy.py script in QIIME. Sequences that did not return significant hits at >90% identify were labeled as “no blast hit” (unassigned), and we attempted to determine their putative identity by using inferences based upon global blast search.

Molecular data were analyzed on the basis of presence/absence of taxonomically assigned MOTUs in each sample, as quantitative data based on the number of sequences ascribed to a given MOTU are unlikely to provide accurate quantitative estimates due to factors other than species abundance that likely interfere with the number of times a sequence was observed (e.g. number of biological cells present in the sediment, DNA density in tissue, primer matching, etc.). For the most accurate comparisons of metazoan diversity across morphological and molecular datasets, analyses were performed with and without the unassigned MOTUs. While the results were congruent, those obtained excluding the unassigned MOTUs are reported here, and results including these MOTUs can be found in the supplemental information section. The decision to exclude unassigned MOTUs was particularly important for the COI dataset, which had the most unassigned (93% of all MOTUs). We determined the putative identity of the unassigned COI and 18S MOTUs with the aid of NCBI’s BLAST stand-alone program [46], using the public nucleotide database implemented with the blastn algorithm at default parameters.

Presence/absence information obtained from the molecular datasets were compared to a quantitative morphological dataset containing 322 species collected and taxonomically identified by the IUEM/REBENT network in 2011, in order to appraise the loss or gain of information obtained with each method in terms of alpha and beta diversity estimates. Given the inherent difficulties of morphologically identifying organisms smaller than 1mm, the morphological dataset was generated from animals with body sizes ≥1mm, while the molecular datasets included meiofauna (animals with body sizes < 1mm [47]) in addition to macrofauna. Therefore, diversity analyses were performed comparing 1) morphological data (≥1mm) with all molecular data (≥ 0.5mm) and 2) morphological data (≥1mm) with molecular data ≥1mm (excluding 0.5mm) for consistency and to better evaluate similarities and differences across survey techniques. A list of the 322 morphologically identified species and their presence/absence in each meadow is shown in S1 Dataset.

Multiple rarefactions were applied to the molecular datasets to obtain standardized estimates of alpha diversity using Chao1 measurement for species richness [48, 49]. Chao1 values were calculated using the *specpool* function of the Vegan community ecology package (v.2.0–10), executed in the statistical software R (v.3.0.2) [50]. Analysis of Similarities (ANOSIM) testing was done to determine if significant differences exist between sampling groups (core, mesh size, meadow), while Principal Component analyses (PCA) were completed to provide spatial illustrations of community structure across meadows, and was performed on the basis of Jaccard distances to minimize the weight given to absence (0) values. Both ANOSIM and PCA analyses were carried out using the software PAST [51]. Finally, taxonomic community compositions by phyla were illustrated via a phylogenetic tree produced using the Interactive Tree of Life iTOL tool [52].

## Results

### Molecular and morphological datasets

The Roche 454 FLX sequencing of *Zostera marina* sediment produced a total of 622,468 and 190,509 raw sequences, which were quality filtered to 412,838 and 153,463 for COI and 18S, respectively (S1 Table). The additional removal of singletons and chimeras produced 411,086 (COI) and 150,677 (18S) sequences, which were then clustered into 13,492 and 1,316 representative MOTUs for COI and 18S, respectively.

Table 2 presents a list of the 50 most common metazoan species recovered and morphologically identified from the sediment of *Z*. *marina* seagrass by the IUEM for the REBENT network. The list details the presence of COI and 18S barcode sequences matching the genera and/or species available in the public sequence database GenBank (NCBI), as well as those families, genera or species that match to MOTU sequences in the environmental metadatasets. At the present, 51% (COI) and 80% (18S) of sequence barcodes of the 50 most common and abundant metazoan species are publically available in NCBI, encompassing 35 families and 48 genera (Table 2). From the COI environmental metadataset, seven MOTUs blasted up to the species level (14% of all common species and 50% of the barcodes added to the COI reference database), and five to the family level. For the 18S metadataset, 14 MOTUs blasted up to the species level (28% of all common species and 42% of the barcodes added to the 18S reference database), seven to the genera and five to the family (Table 2).

### Uncovering the identity of taxonomically unassigned MOTUs

“No blast hit”, or unassigned matches, recognized an inability of the BLAST method implemented through QIIME to uncover a close sequence matching the target with an identity of >90%.

**Fig 3:**
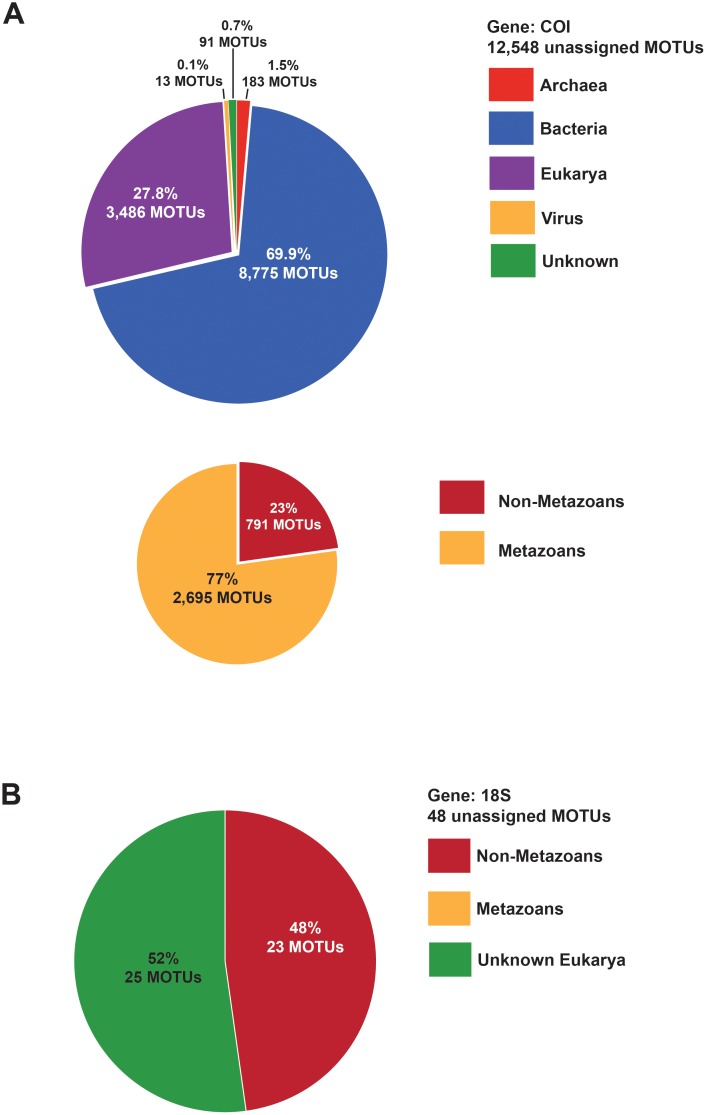
Taxonomic percentages and counts of Molecular Operational Taxonomic units (MOTUs) initially unassigned for COI and 18S, inferred using NCBI BLAST public nucleotide database. (A) Taxonomic percentages for the 12,548 unassigned MOTUs for the COI gene. The top pie chart illustrates the proportions of each domain, while the bottom pie chart estimates the proportions of metazoan versus non-metazoans within the eukaryotes. (B) Taxonomic percentages for the 48 unassigned MOTUs for the 18S gene. The pie chart is divided into non-metazons versus unknown Eukaryotes. The initially unassigned MOTUs for both genes were identified using the blast-stand alone program with the “blastn” algorithm under default parameters.

Of the 13,492 MOTUs uncovered for COI, 944 were assigned to metazoans, while 12,548 (93%) remained unassigned. To glean the identity of the unassigned MOTUs, we performed the blastn procedure for each unassigned sequence against NCBI’s global public database with the aid of the stand-alone BLAST command line program. The overall findings identified the closest match for 1.5% of the MOTU sequences as Archaea, 69.9% as Bacteria, 27.8% as Eukarya, 0.1% as viruses and 0.7% as still unknown/unidentified with no match in the public database (Fig. 3A, S2 Dataset). Further analysis of these 0.7% unknown revealed open reading frame interruptions, suggesting the amplification of pseudogenes, possibly “*numts*”, or nuclear copies of mitochondrial derived genes that become non-functional/coding and are often seen in COI [53, 54] (Fig. 3A). As the blastn algorithm can be less stringent than the BLAST program implemented in QIIME, we cannot discard the occurrence of *numts* among the MOTUs unassigned after QIIME BLAST but assigned after the blastn procedure. Therefore, while *numts* are expected to fall close to their original mitochondrial sequence, we detail only analyses with the 944 initially assigned MOTUs below. We did, however, compare diversity results including and excluding the initially unassigned 12,548 MOTUs (S2 Table, S3 Table, S4 Table, S5 Table, S1 Fig.). The results for both datasets showed similar qualitative results for community structure, although diversity estimates are lower bounded when excluding the initially unassigned MOTUs.

Of the 1,316 MOTUs uncovered for 18S, 1,174 were identified as metazoans and 94 as non-metazoans, while 48 (3.5%) remained unassigned, which is a relatively low percentage compared to what has been observed previously (10%, [55]). To glean the putative identity of the 48 unassigned MOTUs, these sequences were also blasted against the NCBI public database, for which we found 48% of the initially unassigned MOTUs matched to non-metazoan eukaryotes, while 52% were still unknown (Fig. 3B, S3 Dataset). Given that the 52% of unknown MOTUs could contain metazoans, we compared diversity results excluding and including unassigned 18S MOTUs, but for the rest of manuscript, we discuss only the MOTUs initially assigned, similarly to what was done for the COI dataset. Both diversity analyses including and excluding the 48 initially unassigned MOTUs also show similar results (S2 Table, S3 Table, S6 Table, S7 Table, S2 Fig., S3 Fig.).

### Taxonomic community composition by phyla

**Fig 4:**
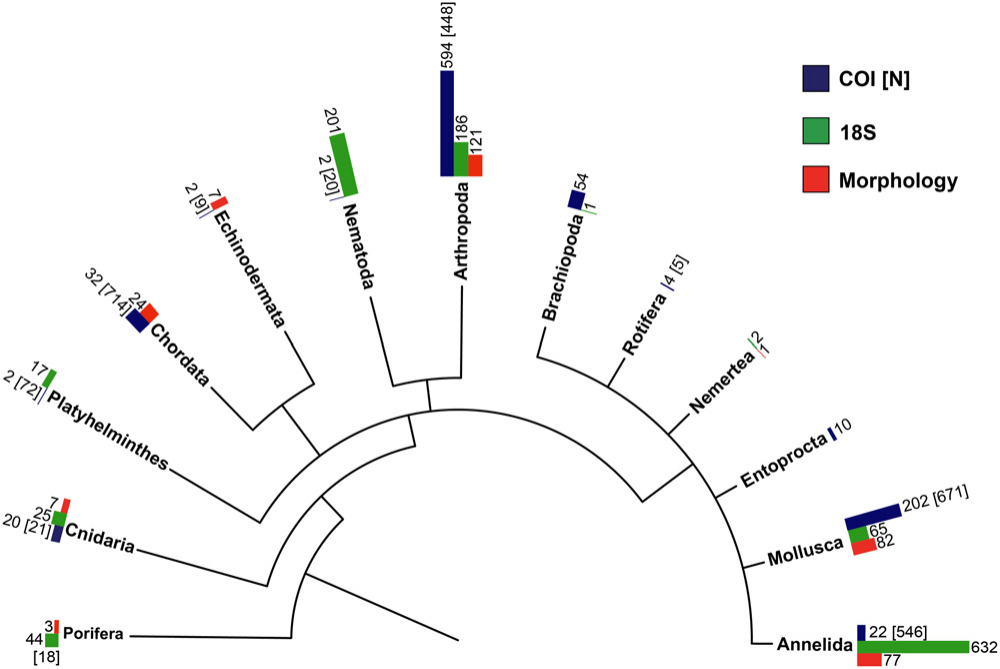
Frequencies of phyla uncovered in *Zostera marina* sediment, using morphological and molecular (COI and 18S) surveys. Bar lengths correspond to the frequency of molecular operational taxonomic units (MOTUs) assigned to a specific phyla for each survey method. The numbers above each frequency bar refer to the number of MOTUs identified by each respective survey method. Numbers in brackets [N] above the COI bars correspond to the number of COI MOTUs that were initially unassigned, but once individually blasted to identify taxonomy, matched to major phyla. Numbers without brackets above COI correspond to those MOTUs that were initially identified (n = 944). Tree was produced using the iTOL tool.

The number and frequency of MOTUs assigned to 13 major metazoan phyla uncovered by morphologic and molecular methods are illustrated in Fig. 4. Colored bar lengths correspond to the frequency of MOTUs assigned to a specific phyla by each survey method, while the numbers above each frequency bar refer to the number of MOTUs identified. Numbers in brackets [N] above the COI bars correspond to the number of COI MOTUs that were initially unassigned, but once individually blasted to identify taxonomy, matched to one of the major phyla. Numbers without brackets above COI correspond to those MOTUs that were initially identified (n = 944). All frequency values can be found in the S4 Dataset. While Annelida, Arthropoda and Mollusca were the most frequently observed phyla with the highest number of MOTUs being assigned to these groups, each method differed in its taxa specific affinity. The COI survey identified arthropods as the most common phylum with and without taking into account the previously unassigned MOTUs, followed by mollusks, whereas 18S had the highest number of MOTUs assigned to annelids followed by arthropods, and revealed fewer molluscan taxa. Both morphological and COI surveys were better for unraveling Chordata and Echinodermata, whereas 18S identified Nematoda and Porifera (sponges). Taking into account previously unassigned COI MOTUs, COI was best at identifying Platyhelmithes (flatworms) and Cnidaria.

**Fig 5:**
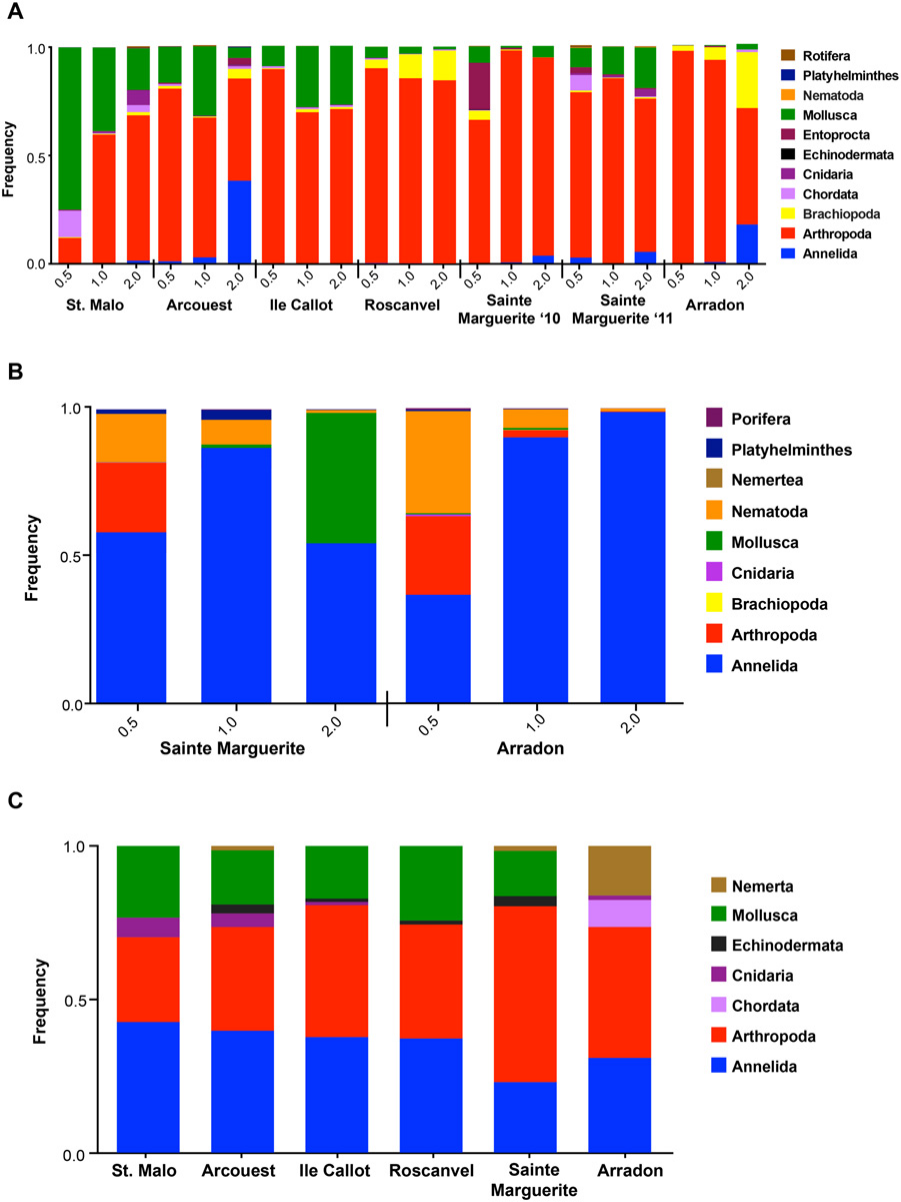
Frequency comparisons of phyla found in three size class fractions 0.5, 1.0, and 2.0 mm. (A) Frequency of phyla by size identified using the COI gene at six meadows. Analyses include two years (2010 and 2010) for the Sainte Marguerite meadow. (B) Frequency of phyla by size identified using the 18S gene at two meadows. (C) Frequency of phyla found at the ≥1 mm size class via morphological identifications.

The frequencies of metazoan phyla uncovered in each of the three mesh size fractions are shown in Fig. 5. The better capacity of COI for uncovering molluscans and arthropods and 18S for annelids and nematods is maintained, regardless of mesh size. For COI, annelids had the highest representation in 2mm at Arcouest and Arradon, while mollusks were detected at all size classes across meadows (Fig. 5A). Nematodes were barely detected using the COI survey, but an unusually high frequency of mollusks and chordates were found in the 0.5mm fraction at Saint Malo. The COI survey also identified mostly arthropods in the 1–2mm fractions of sediment at Sainte Marguerite in 2010 whereas a predominance of mollusks was revealed one year later in the same quadrate, a pattern that was consistent across core replicates.

For 18S, nematodes were dominant in the 0.5mm fraction, and their presence apparently decreases with larger size classes at both meadows analyzed (Fig. 5B). Arthropods were also highly represented in 0.5mm, while mollusks were mostly detected in the 2mm size class at Sainte Marguerite. At Arradon, the larger size classes appear heavily dominated by annelids. Lastly, the morphological survey consisted of ≥1mm size fractions and identified a nearly even frequency of arthropods and mollusks at all meadows except the southernmost Arradon (Fig. 5C). At Arradon, representatives from Chordata and Nemertea appeared as frequent macrofauna, whereas mollusks were not detected. Overall, these results support the use of size filtering for unveiling an all-encompassing picture of community biodiversity.

### Alpha diversities and community comparisons across spatial scales

**Fig 6:**
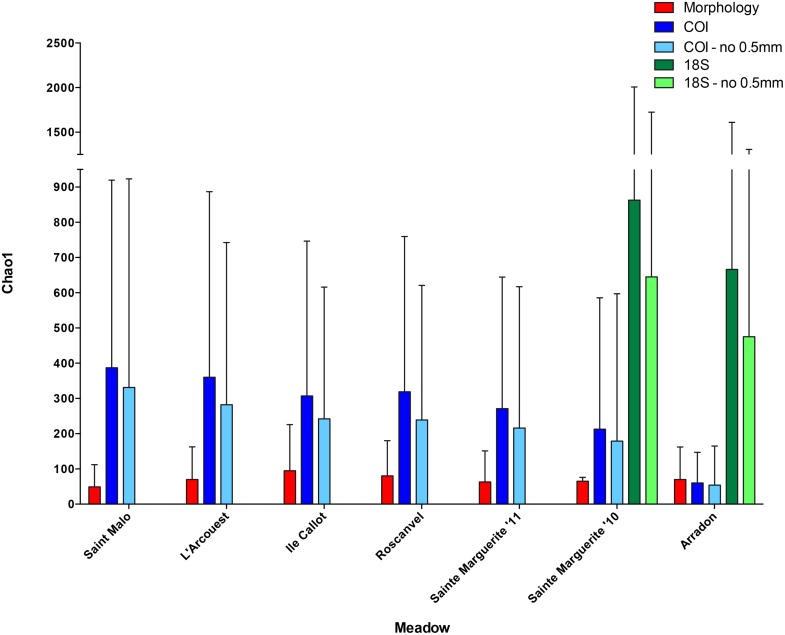
Chao1 values presented by meadow for morphological and molecular surveys. Dark bar colors for the molecular surveys denote the inclusion of three size fractions, while light bar colors denote the exclusion of the 0.5mm size fraction, done for accurate comparisons with morphological surveys. Chao1 values were calculated using Vegan community ecology package implemented in R [50].

As the morphological survey consisted of only macrofauna, for the purposes of accurate comparisons, alpha diversity analyses were performed against molecular surveys including and excluding the 0.5mm fraction. Both molecular datasets consistently exhibited much higher values (mostly five to ten times higher) than that of morphology, and when including only assigned MOTUs for the propose of being conservative, 18S exhibited much higher Chao1 values for Sainte Marguerite and Arradon compared to COI (Table 3 and Fig. 6, see S5 Dataset, S6 Dataset and S7 Dataset for all diversity values). Additionally, comparisons of alpha diversity by meadow did not show strict correlation across morphological and molecular surveys (Fig. 6, S8 Table), with meadows having the highest species richness values differing depending on the survey method implemented. In general, these analyses illustrated that even when excluding the 0.5mm fraction, molecular surveys deliver much higher diversities when compared to the morphological survey.

**Table 3:** Chao1 values by *Z*. *marina* meadow for each survey method. The value after the +/- symbol indicates the standard error.

Meadow	Method				
	Morphology	COI		18S	
		Including 0.5mm	Excluding 0.5mm	Including 0.5mm	Excluding 0.5mm
Saint Malo	49 ± 63	387 ± 497.30	331 ± 555.12	---	---
L’Arcouest	70 ± 92.78	360 ± 506.60	282 ± 474.53	---	---
Ile Callot	95 ± 131	307 ± 460.12	243 ± 351.93	---	---
Roscanvel	80 ± 100.34	319 ± 439.19	240 ± 339.01	---	---
Sainte Marguerite 2011	63 ± 63	271 ± 366.60	216 ± 351.84	---	---
Sainte Marguerite 2010	65 ±11.22	213 ± 392.56	179 ± 417.34	863 ± 1144.43	645 ± 1075.27
Arradon	70 ± 92.22	60 ± 77.79	54 ± 116.23	666 ± 914.06	475 ± 806.2

ANOSIM testing for differences in community structure across spatial scales revealed that for both morphological and molecular methods, meadows are significantly different from one another (p ≤ 0.013), while cores are not significantly different within meadows (p ≥ 0.891, Table 4). For molecular analyses, mesh sizes also differed in terms of community composition (COI, p = 0.001, 18S, p = 0.014), with the 0.5mm fraction being highly dissimilar from both of the larger size classes (pairwise p ≤ 0.03, S4 Table, S5 Table, S6 Table, S7 Table). When results from the three mesh sizes were pooled to assess biodiversity present by core, cores were found to be homogeneous in terms of community composition within each meadow (COI, p = 0.945, 18S, p = 0.891, S3 Table). This result is in line with homogeneity also observed among morphologically described cores (S9 Table and S10 Table), supporting a lack of significant spatial heterogeneity that advocates the role of cores as replicates in the sampling scheme.

**Table 4:** Analysis of similarities (ANOSIM) testing across meadow, core and mesh size spatial scales, performed in PAST [51]. NS = not significant, * 0.5mm size fraction significantly differs from both 1 and 2mm fractions (p < 0.05).

	Method		
Taxonomic community assessment	COI	18S	Morphology
Ho: meadows do not differ	p = 0.001	p = 0.013	p = 0.001
Ho: cores do not differ	NS	NS	NS
Ho: mesh sizes do not differ*	p = 0.001	p = 0.014	---

### Morphological and molecular multidimensional analyses

**Fig 7:**
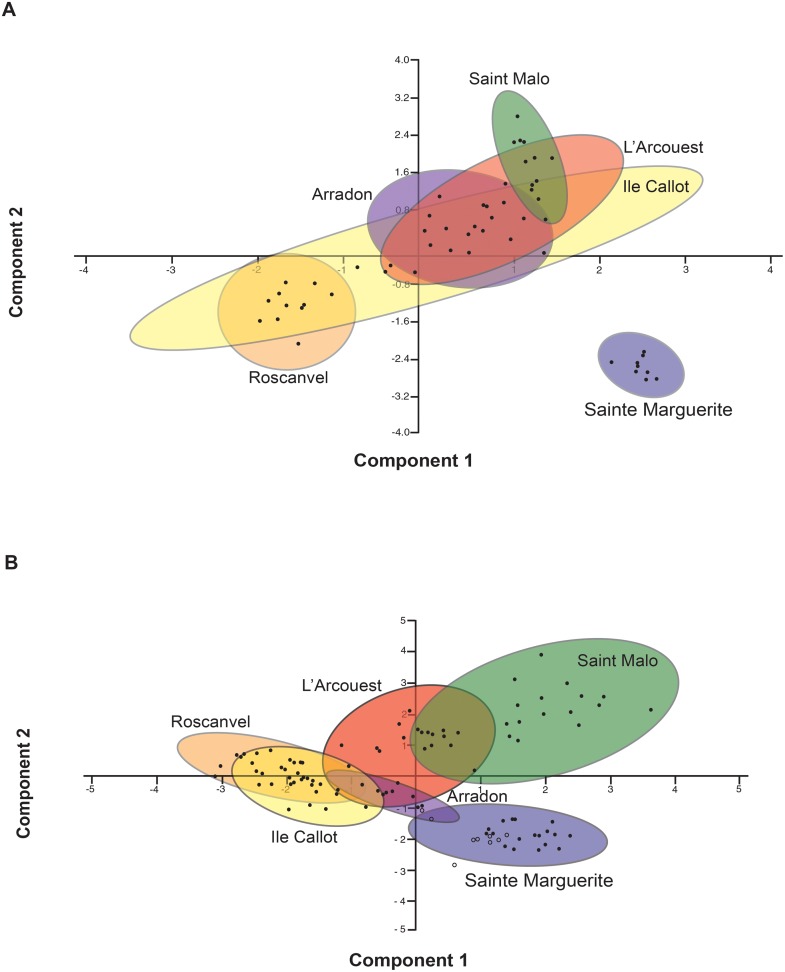
Principal Component Analyses (PCA) for the morphological and molecular surveys of meadows. (A) PCA for the morphological inventory. (B) PCA for molecular inventory was performed using the COI gene. Each PCA was implemented using Jaccard dissimilarity matrices. Points are individual sediment samples taken at a specific core and mesh size, while colored ellipses are 95% confidence intervals for each meadow. For COI, the Sainte Marguerite cluster contains samples from 2010 (open circles) and 2011 (closed circles). Colors of the ellipses are specific to each meadow and these colors were maintained across both PCAs.

Principal Components Analyses (PCA) of each survey identified similar patterns of community composition (Fig. 7). The six meadows emerged as six distinct clusters supported by significant results from the ANOSIM analyses of both morphological and molecular (COI) surveys (Table 4), and the PCA analyses show the meadows arranged in a similar orientation for both morphological and molecular surveys (Fig. 7). Furthermore, Sainte Marguerite was defined by both methods as having a most dissimilar community composition when compared to the other meadows, which is likely the result of both oceanographic forces and demographic history of this specific meadow. Despite these similarities across surveys, mantel testing using Jaccard beta diversity distances for comparison of morphological and molecular surveys showed non-significant results (p > 0.05, S8 Table).

Even with significant differentiation of the six clusters corresponding to each meadow via ANOSIM testing, the molecular COI PCA illustrated overlap between neighboring meadows of Roscanvel and Ile Callot, as well as Saint Malo and L’Arcouest (Fig. 7B), similar to that obtained on the basis of the morphological survey. Stronger divergence of meadows was depicted with the COI survey, identifying samples clustering to their specific meadow, irrespective of core collection or mesh size.

For the molecular survey of Sainte Marguerite, samples from 2010 (denoted by open circles within the cluster) and samples from 2011 (denoted by closed circles within the cluster) grouped together, suggesting that overall temporal community structure remained globally similar across 2010 and 2011 for this specific meadow. Finally, apart from Sainte Marguerite and Arradon, COI meadow community structure depicted an east/west gradient across the region beginning with Saint Malo, L’Arcouest, Roscanvel and Ile Callot.

## Discussion

This study presents one of the first comparative analysis of inventories based on a dual morphological and molecular approaches for the same sampling sites, in addition to the inclusion of replicates to ensure statistically supported results. DNA metabarcoding of *Zostera marina* seagrass sediment uncovered vast taxonomic diversity, revealing that in a single snapshot, the invertebrate metazoan community diversity estimated by molecular surveys consisting of hundreds to thousands of MOTUs largely exceeded the 322 species uncovered by the morphological survey. In addition to this enhanced power of molecular barcode for unraveling the diversity of organisms present in the community, dissimilarities in the ranking of alpha diversities among meadows were also recorded (Fig. 6). This clearly illustrates the non-exhaustive nature of inventories obtained with both methods and their only partial overlap, confirming, despite the use of replicates, the extensive occurrence of false negative [56]. These differences, despite the comparison within the exact same locations, may stem from several reasons that are discussed more extensively below, among which the fact that each method requires samples of a different nature as a result of technical constraints. However, a congruent picture of community structure (beta diversities) was illustrated through spatial analyses, which identified statistically supported clusters corresponding to each meadow with both survey approaches, including the remote position of the highly differentiated Sainte Marguerite meadow community (Fig. 7).

The methods and results reported here provide another step towards the development of a powerful, repeatable and cost effective metabarcoding protocols for baseline studies integrated within environmental assessments while still pointing towards some required improvements. When compared to the morphological inventory, the observations of high diversity and the lack of some common species in the molecular inventory, as well as the amount of unknown MOTUs roughly assigned to related taxa, makes metabarcoding a powerful, but still partly blind method. Specifically, our difficulties to uncover a higher percentage of the most common species underlines i) the necessity of multi-gene surveys, ii) the further improvement of sampling protocols to allow the comparison of technically more similar samples between survey methods (rather than distinct sample collections from the exact same quadrates) and to include larger scale spatial and temporal sampling, as well as iii) synergistic efforts between teams involved in molecular and morphological taxonomic identifications to feed, standardize and curate reference libraries. Additionally, the systematic molecular barcode of museum holotypes are already being performed at various museums around the world [57]. Awaiting the results of this global improvement, we advocate a double approach (the thorough comparison of morphological and molecular inventories using “double described” species) at least during the first phase of biodiversity assessments, before safely engaging in longer-term ‘blind’ metabarcoding surveys.

### Advantages and disadvantages of both survey approaches

Advantages and disadvantages of both morphologic and molecular identification approaches have been discussed in detail recently [24,26,43]. A notable advantage of morphological screenings includes the ability to view whole specimens and to differentiate between biological footprints of transient organisms and those contemporarily present into the community. Further, the details provided by morphological screenings of organisms provide better taxonomic identifications for particular lineages. Morphological inventories also provide actual species abundance counts, whereas metabarcoding datasets are still limited to presence/absence data, given that technical factors likely influence the number of times a sequence is observed more than the abundance of the corresponding taxa [24].

The disadvantages of morphological approaches are amplified by dwindling taxonomic expertise and include difficulties with resolving cryptic species or meiofauna, as well as the complications of generating standardized protocols for assessment of broad community biodiversity. The time costs associated with taxonomic identifications has led to investigations for more time and cost efficient approaches for rapidly assessing ecosystem community structure [33]. Molecular methods, such as metabarcoding, have taken the forefront as solutions to the present dilemma, as these techniques have the ability to generate more extensive biological inventories while providing researchers with a standardized and economical approach to expeditiously assess community structure and biodiversity. Moreover, molecular collections taken with respect to organisms with body sizes less than 1mm can reveal species diversity that likely play a significant role in the functioning and stability of target communities, but is often difficult to retrieve via morphologic methods alone.

In the present study, comparisons of molecular and morphological surveys using only those samples obtained at ≥1mm size fractions show molecular methods unmasking a higher amount of invertebrate diversity, providing a broader view of the taxa present in the community. We note that comparing only ≥1mm size fractions provides a conservative estimate of diversity, however the finding of higher diversity is at least partially due to the enhanced ability of molecular methods to unmask the diversity of Annelida, which represent the dominant group of marine macrofauna [58]. Further, molecular surveys were particularly adept at uncovering animals common in smaller class sizes, such as nematodes (Fig. 5B), in addition to identifying “temporary meiofauna”—eggs or juveniles of larger animals found in the mesh size <1mm [59] (Fig. 5A). Moreover, metabarcoding surveys may include the transitory presence of individuals having left biological footprints through partial remains such as scales, mucus, spines and feces. In some cases, mobile species native to the community might escape morphological surveys, as in the case of gastropods (such as those MOTUs found matching *Dendronotus frondosus*, *Goniodoris nodosa* and *Archidoris pseudoargus*), which were recorded in the molecular inventory but were not present in the morphological survey. It may be argued that molecular inventories could erroneously include incidental species (non-native transient diversity), however, the systematic inclusion of spatial replicates in the form of cores allows checking for the consistency of environmental collections.

### The synergistic relationship between molecular and morphological identifications

Molecular surveys (a combination of both genes) uncovered 36% common species in a single survey, suggesting that the other 64% were either i) not present in the sediment sampled at that particular snapshot in time, ii) were present, but the tissue contained degraded DNA, iii) were not amplified with the primers employed, or iv) were not recognized through the assignment as adequately homologous to taxa present in reference databases. We also note that the low number of common species barcodes we obtained to add to the reference databases (14 for COI and 33 for 18S) could be due to mismatch in the primers or low quality of DNA from morphologically identified specimens that had been preserved part time in formaldehyde, which may have impaired the retrieval of common taxa among inventories.

The implementation of non-specific or “too-specific” primers could explain why the majority of common metazoans were not found. In the present study, we tested and implemented universal and degenerate primers for both COI and 18S, in order to increase our chances of retrieving the widest array of taxa possible. Despite our initial primer testing, the amplification of *numts*/pseudogenes and primer mismatching remain characteristics of the markers and technologies and we continue to work towards the improvement of these tools. Given the inherent characteristics of differing DNA types (evolutionary rate, neutrality, and presence of introns, for example), previous authors have suggested the use of a multilocus barcoding approach that includes mitochondrial and nuclear genes, to minimize gene/primer specific biases and enhance resolution at different taxonomic levels, a complementarity clearly depicted in Fig. 3 [15,60,61]. Another reason for the lack of common species uncovered could be due to the analyses of only 10g of sediment taken from the sieved samples. Though our initial goal was to identify species present in a snapshot of sediment at a given time, replicate testing of sediment within each sample for consistency may determine if more species were present in the overall sample. However, in an analysis of earthworm communities, no differences were found between distinct DNA extractions performed on the same environmental sample [56].

Prior research described the problem of deriving accurate taxonomy using current databases, given the low reliability of publicly available taxonomic assignments past the class or family level, especially for organisms in smaller class sizes [6,25,26,43]. As our sequence barcodes were taxonomically identified prior to being added to the reference database libraries, the inability to detect some of the common species if indeed present in the sediment, is not likely due to curation issues or misidentifications in the libraries used for assignments but more likely to technical pitfalls described above (the quality of DNA or primer matching). However, given metabarcoding’s reliance upon reference libraries for taxonomic assignments, our results also bring to light additional evidence for the absolute need for properly curated (i.e. taxonomically and molecularly identified) libraries to yield a higher likelihood of correct taxonomic assignment. This is the required condition to obtain inventories that are not only quantitatively superior for work on the spatial and temporal distribution of diversity, but also qualitatively reliable for understanding the taxonomic composition of communities and their evolution under differing environmental conditions.

We therefore suggest that pilot studies, including parallel morphological and molecular surveys, should be performed to i) formally test the efficiency of metabarcoding using the best protocols and genetic markers for the ecosystem studied, ii) provide a baseline for a temporal survey that could implement the blind metabarcode method once taxonomic coverage and assignment are enhanced as well as the number of replicates determined for a reliable comparison, and iii) provide molecular databases against which future blind metabarcode datasets could be compared, in order to survey the temporal evolution of a representative subset of the community. This would allow a baseline strategy with necessary parameters determined, prior to performing fully “blind” metabarcode studies. Ultimately, refining the reference dataset to target specific groups would be useful in ecosystem investigations where the goals are to provide diversity estimates and community composition to the genus or species level based on reliable taxonomic inferences.

### Differences in relative alpha diversities

The discrepancy between the relative alpha diversities seen with the morphological and metabarcode inventories across the different meadows is contrary to what has previously been observed [24]. This cannot be attributed to the inclusion of the meiofaunal compartment at constant “time costs” in the metabarcode inventory, as similar results were obtained when including or excluding the smaller mesh size. Another explanation might be the nature of the collection methods and taxonomic indices upon which each approach relies. The morphological survey was performed using several collection devices and depended upon the presence of complete specimens, while the metabarcoding approach relied upon cores and typically identifies everything present in the environmental sample, including the remains of transient individuals. At first glance, the exposure of a community to transient individuals might prevent the accurate molecular assessment of true community structure, when visual surveys (i.e. morphological identifications) inherently filter transients and partial remains. However, metabarcoding assignments confirm that multiple cores result in similar community compositions within the six seagrass meadows, a finding that matches the morphological survey and supports the lack of spatial heterogeneity at the meadow scale. These findings suggest that each core can be considered a replicate for a representative description of communities.

Alpha diversity comparisons by meadow lacking strict correlation across surveys could also be the result of the variation in community composition in terms of phyla identified by molecular surveys (Figs. 4 and 5), which is magnified by the capacity of the molecular genes to uncover specific taxonomic groups. The 18S gene was particularly useful for identifying annelid polychaetes, a group which is known to dominate marine macrobenthos inventoried thus far [58], but is less thoroughly characterized by morphological surveys. The morphological survey showed high performance for identifying larger size class animals belonging to the phyla Chordata and Echinodermata, while underrepresenting phyla that can contain groups of smaller size classes (i.e. Annelida, Nematoda). Although this metabarcoding approach includes two types of markers to provide a more comprehensive estimates of biodiversity based on single surveys, the dominance of Annelida and Arthropoda provided by 18S and COI, respectively, likely had a large impact on the alpha diversities (Fig. 6).

The factors described above provide potential reasons for the observed differences in alpha diversity estimates of morphological versus molecular surveys, and the stronger power to uncover a broader range of taxa exhibited by the molecular surveys likely provide a more representative community inventory, despite a major disadvantage of not having species counts and thus quantitative data available.

### Comparison between COI and 18S barcoding markers

Results identified the18S gene as uncovering higher numbers of common species as well as greater species diversity at the Sainte Marguerite and Arradon meadows, when compared to COI. We do not suggest that 18S is a unilaterally better marker, but in this case, had a better curated reference database and likely better primer matching owing to the composition of the communities studied, which were characterized by a high prevalence of taxa having better affinity to 18S primers. Further, we consider that the shorter fragment targeted by 18S (450bp versus 710bp with COI primers) may also explain a higher success when DNA quality is limited as with environmental samples. Both COI and 18S surveys still exhibited different and somehow complementary taxonomic specific biases, which pleads for multi-gene barcoding approaches. Future studies on different communities will continue to shed light on the intrinsic quality of each marker to unveil the taxonomic composition of a community as a whole, and performing inventories using additional/new markers and primers will enhance complementarity with current markers and limit taxonomic bias.

### Community structure in morphological vs. molecular surveys

A similar pattern of clustering and meadow based community differentiation obtained with both survey methods was illustrated through multidimensional analysis (Fig. 7). While we do find some overlap of taxa within the seagrass communities, the stronger clustering of meadows shown by the molecular survey is likely the result of the weight given to rare taxa via the implementation of presence/absence analyses. Additionally, the molecular survey did identify higher levels of biodiversity than the morphological survey, and this more complete inventory provided a more holistic picture of community structure at *Z*. *marina* meadows. Furthermore, the analyses of 18S datasets from Sainte Marguerite and Arradon in 2010 also identify community differentiation amongst these two meadows (S2 Fig. and S3 Fig.). This discovery also provides confidence for temporal patterns and additional support for the reliability and repeatability of metabarcoding inventories for community analyses.

The remote position of the Sainte Marguerite communities was evident with both molecular and morphological approaches. The distinctiveness of the Sainte Marguerite community is likely due to more frequent disturbances in relation to its exposure to stronger oceanographic forces and patch extinctions. Sainte Marguerite is the least sheltered meadow that receives the highest wave energy as it is directly facing west, and its exposition to open sea currents may also explain a higher temporal variability. In 2008, local extinctions of large patches of this meadow were recorded, followed by progressive recolonization [35,62]. These demographic vagaries may be a result of oceanic influence and in turn impact the diversity and stability of associated communities. Interestingly, as sediment samples for this meadow were taken in 2010 and 2011, community composition appears to have changed only slightly over the course of a year, and more temporal studies may uncover whether the changes will continue with time elapsed since the last demographic crash.

## Conclusions

Results presented here support metabarcoding as an powerful and repeatable approach for expeditiously and cost effectively uncovering community structure and higher species richness than morphological methods, underlining the added value of the multi-gene approach to obtain a more balanced picture of represented taxa and overall community composition. The testing of a protocol that allowed replicate core sampling and analyses of specimens of distinct size classes to provide a more accurate view of community diversity illustrated that metabarcoding is consistent. Additionally, results raise substantial warnings as to the need for more direct comparisons, as well as comprehensive and rigorously curated reference libraries in order to shed light on the taxonomic identity of MOTUs detected through blind metabarcode. Results suggest that a first step consisting of “double inventories” (morphological and molecular) using standardized protocols may be required, in order to check the accuracy of molecular protocols for the targeted communities, in addition to supplying a reference inventory and molecular database for subsequent “blind metabarcode” temporal surveys.

## Supporting Information

S1 Dataset Morphological survey of 322 species found at six *Zostera marina* seagrass meadows in 2011, identified by IUEM/REBENT. Each meadow contains collections from three cores, and presence of species is indicated by “1”, while absence is indicated by “0”. The most common species found in this inventory are highlighted in yellow, while those common species *not* found in the inventory are highlighted in light green. Columns for those species uncovered by each gene are highlighted in dark green for 18S and dark blue for COI.
(XLSX)

S2 Dataset Global BLAST results for 12,548 unassigned COI MOTUs. Included are MOTU names, the GenBank identifying number (GI), the e-value, the closest species match, the kingdom, the phylum (for Eukarya only), and the frequencies of each kingdom present in the dataset. The blastn algorithm was implemented under default parameters (see Appendix C of the BLAST command line applications user manual, http://www.ncbi.nlm.nih.gov/books/NBK1763/).
(XLSX)

S3 Dataset Global BLAST results for 48 unassigned 18S MOTUs. Included are MOTU names, the GenBank identifying number (GI), the e-value, the closest species match, the kingdom, the phylum, and the frequencies of each kingdom present in the dataset. The blastn algorithm was implemented under default parameters (see Appendix C of the BLAST command line applications user manual, http://www.ncbi.nlm.nih.gov/books/NBK1763/).
(XLSX)

S4 Dataset Frequencies of taxa identified by metazoan phyla by each respective survey method. (XLSX)

S5 Dataset Chao1 (alpha diversity) and Jaccard distances (beta diversity) for morphological survey inventoried by REBENT. Chao1 values were generated by meadow site using Vegan in the software R, while Jaccard distances were generated by meadow, by core and by sample using the program PAST.
(XLSX)

S6 Dataset Chao1 (alpha diversity) and Jaccard distances (beta diversity) for the COI gene molecular inventory. Chao1 values were generated by meadow site using Vegan in the software R, while Jaccard distances were generated by meadow, by core and by sample using the program PAST.
(XLSX)

S7 Dataset Chao1 (alpha diversity) and Jaccard distances (beta diversity) for the 18S gene molecular inventory. Chao1 values were generated by meadow site using Vegan in the software R, while Jaccard distances were generated by meadow, by core and by sample using the program PAST.
(XLSX)

S1 Fig (unassigned MOTUs included) Principal Components Analysis (PCA) using the Jaccard coefficent, for six meadows described with the COI gene. (PDF)

S2 Fig (unassigned MOTUs included) Principal Components Analysis (PCA) using the Jaccard coefficent, for Sainte Marguerite and Arradon meadows described with the 18S gene. (PDF)

S3 Fig (unassigned MOTUs excluded) Principal Components Analysis (PCA) using the Jaccard coefficent, for Sainte Marguerite and Arradon meadows described with the 18S gene. (PDF)

S1 Protocol DNA extraction and sequencing protocols for REBENT identified metazoans. (DOCX)

S2 Protocol QIIME scripts used for bioinformatics data processing analyses. (DOCX)

S1 Table Characteristics of Roche 454 FLX produced amplicons before and after filtering stages. (DOCX)

S2 Table (unassigned MOTUs included) One and two-way ANOSIM results implemented in PAST under the Jaccard similarity index for 18S and COI under *n* = 9999 permutations. (DOCX)

S3 Table (unassigned MOTUs excluded) One and two-way ANOSIM results implemented in PAST under the Jaccard similarity index for 18S and COI under *n* = 9999 permutations. (DOCX)

S4 Table (unassigned MOTUs included) One-way ANOSIM pairwise matrix p-values for meadow and mesh size in COI. SMG = Sainte Marguerite.
(DOCX)

S5 Table (unassigned MOTUs excluded) One-way ANOSIM pairwise matrix p-values for meadow and mesh size in COI. SMG = Sainte Marguerite.
(DOCX)

S6 Table (unassigned MOTUs included) One-way ANOSIM pairwise matrix p-values for meadow and mesh size in 18S (DOCX)

S7 Table (unassigned MOTUs excluded) One-way ANOSIM pairwise matrix p-values for meadow and mesh size in 18S (DOCX)

S8 Table Mantel test results implemented in PAST for morphology and molecular (COI) Jaccard distances by meadow, under the Bray-Curtis index and *n* = 9999 permutations. Results with unassigned COI MOTUs included and excluded are reported
(DOCX)

S9 Table One and two-way ANOSIM results implemented in PAST under the Jaccard similarity index for the morphology dataset under *n* = 9999 permutations. (DOCX)

S10 Table One-way ANOSIM pairwise matrix p-values for meadows in the morphology dataset. SMG = Sainte Marguerite.
(DOCX)
