# Supplementary figures and images for: Metabarcoding Is Powerful yet Still Blind: A Comparative Analysis of Morphological and Molecular Surveys of Seagrass Communities
Source: PLoS One. 2015 Feb 10;10(2):e0117562. doi: 10.1371/journal.pone.0117562 (PMC4323199; doi:10.1371/journal.pone.0117562)

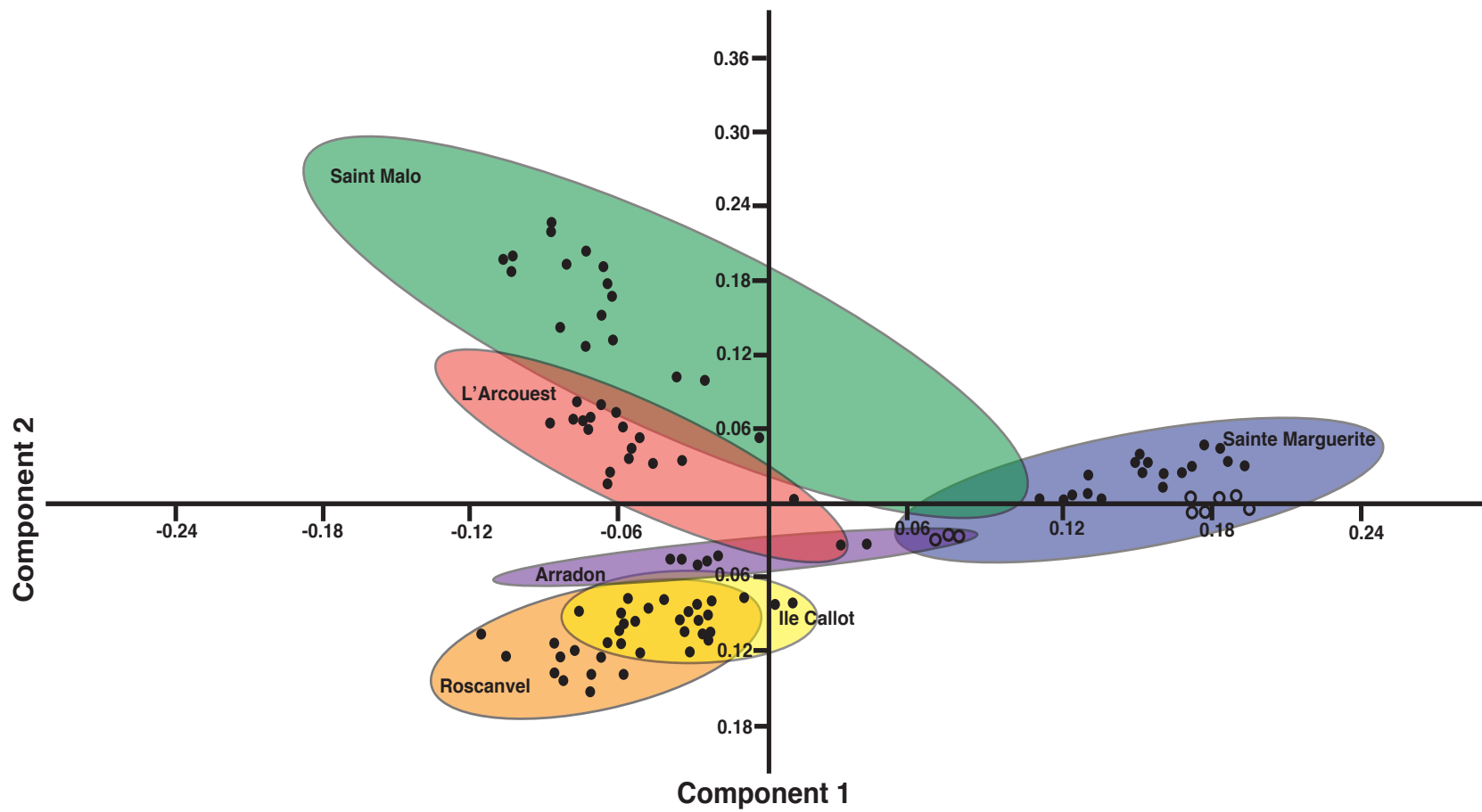

Supplement: S1 Fig — (PDF) [file pone.0117562.s008.pdf]

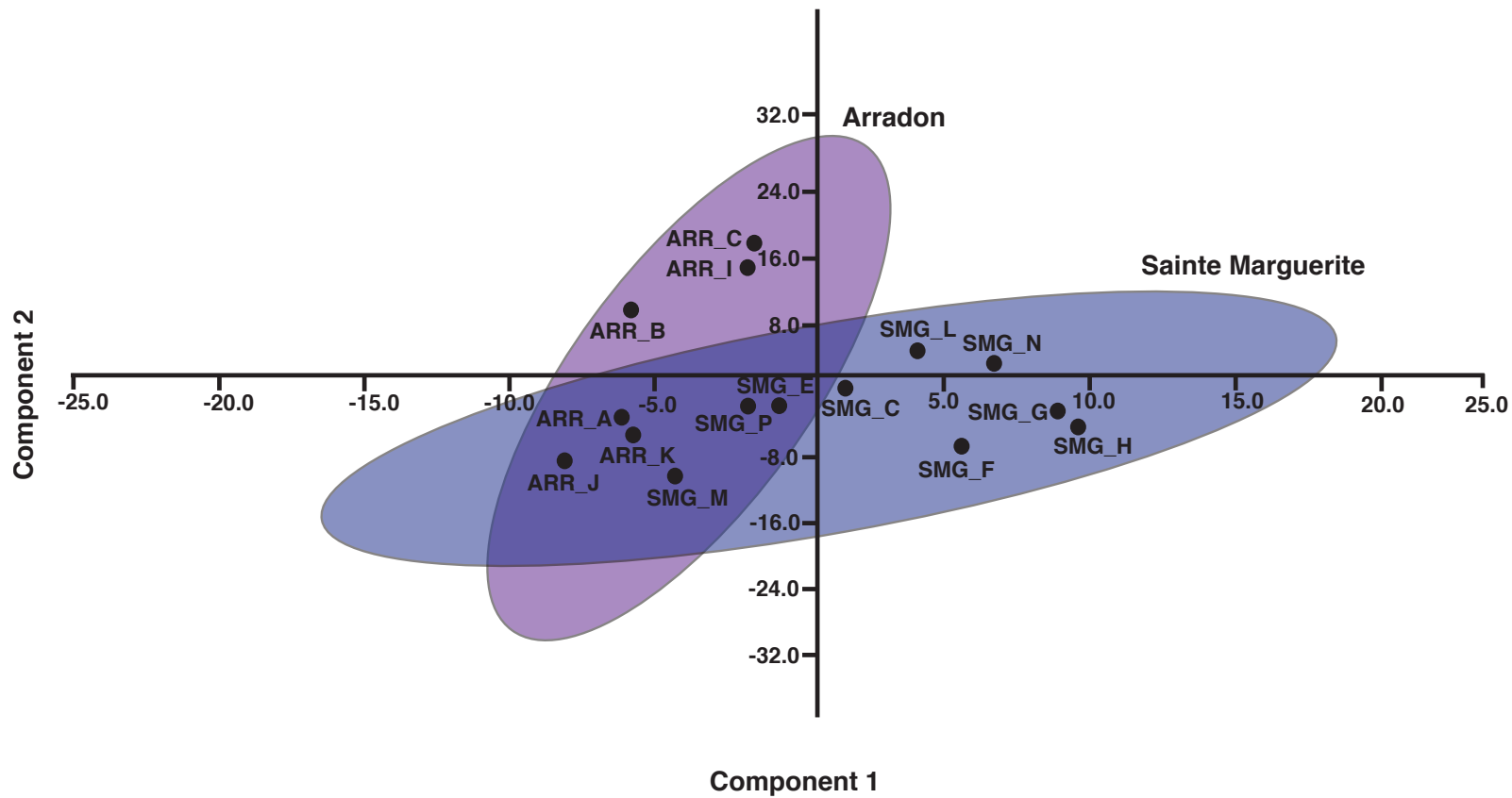

Supplement: S2 Fig — (PDF) [file pone.0117562.s009.pdf]

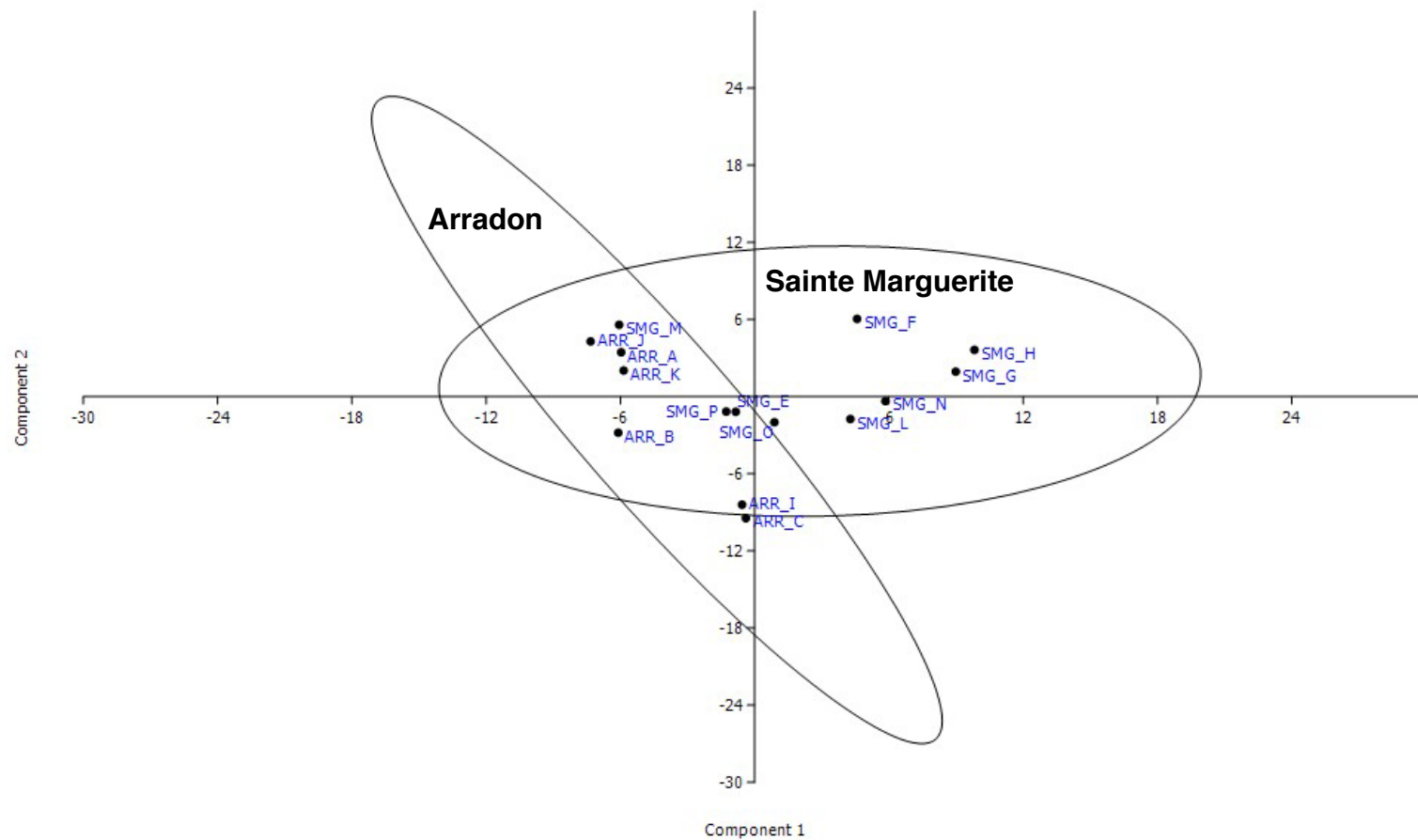

Supplement: S3 Fig — (PDF) [file pone.0117562.s010.pdf]
