# Supplementary material for: Metabarcoding Is Powerful yet Still Blind: A Comparative Analysis of Morphological and Molecular Surveys of Seagrass Communities
Source: PLoS One. 2015 Feb 10;10(2):e0117562. doi: 10.1371/journal.pone.0117562 (PMC4323199; doi:10.1371/journal.pone.0117562)
Supplement: S1 Protocol — (DOCX) [file pone.0117562.s011.docx]

**S1 Protocol**

*DNA extraction*

Whole genomic DNA was extracted according to the CTAB/chloroform protocol ([Doyle 1987](#_ENREF_13)) with the following modifications: tissues were digested in CTAB buffer augmented with 5μl of proteinase k 0.1mg/ml (> 6U/μl) at 60 °C for approximately 2h. At the final stage of the extraction, genomic DNA pellets was cleaned with 70% ethanol and re-suspended in 50 μl sterile water.

*DNA sequencing*

PCR reactions consisted of a total volume of 50μl, which included 0.5 uM of each primer, 1mM of each dNTP, 0.05U/μl of Promega GoTaq^®^, 1x GoTaq Buffer, 2.5mM MgCl_2_, and 1μL of 1/100 diluted DNA template. Amplification was performed using the following PCR conditions: 95 °C (1 min), 35 cycles of 95 °C (1 min), 50 °C (1 min) and 72 °C (1 min), followed by a final elongation step of 72°C (7 min). All PCR products were visualized on 1% agarose gels stained with ethidium bromide. Final PCR products were sent to Macrogen^®^ Europe for purification and sequencing.

*References*

Doyle JJ, Doyle, J.L. (1987) A rapid DNA isolation procedure for small quantities of

fresh leaf tissue. *Phytochemical Bulletin* **19**, 11 - 15
