# Supplementary material for: Metabarcoding Is Powerful yet Still Blind: A Comparative Analysis of Morphological and Molecular Surveys of Seagrass Communities
Source: PLoS One. 2015 Feb 10;10(2):e0117562. doi: 10.1371/journal.pone.0117562 (PMC4323199; doi:10.1371/journal.pone.0117562)
Supplement: S2 Protocol — (DOCX) [file pone.0117562.s012.docx]

**S2 Protocol**

1. check_id_map.py (use original 454 fna/qual files and metadata file)

2. split_libraries.py (removes short and low quality sequences, adds barcodes names) 🡪 seqs.fna

3. identify_chimeric_seqs.py (input seqs.fna and 454.fna [to ID chimeras based on parent sequences] files) 🡪 chimeras.txt

4. pick_otus.py (input seqs.fna to pick otus denovo, without reference file) 🡪 seqs_otus.txt

5. pick_rep_set.py (input seqs_otus.txt; this step is usually done after denovo otu picking and *not ref otu picking*) 🡪 rep_set.fna

6. assign_taxonomy (input rep_set.fna; need reference database [fasta or fna] and taxonomy id [txt] files to assign. Default BLAST method is used for taxonomy assignment) 🡪 rep_set_tax_assignment.txt

7. make_otu_table.py (input rep_set_tax_assign.txt. This otu table includes taxonomic assignments) 🡪 otu_table.biom

8. filter_otus_from_otu_table.py (input otu_table.biom. Singleton removal; this filters sequences appearing < 3 times if –n 3 is passed) 🡪 otu_table_no_singletons.biom

9. filter_otus_from_otu_table.py (input otu_table_no_singletons.biom and chimeras.txt [step 4]. Removes chimeras)

🡪 otu_table_no_chimeras.biom

10. add_metadata.py (input otu_table_no_chimeras.biom and a txt file containing information to add (i.e. accession number for taxonomy assignments) 🡪 otu_table_metadata.biom

11. split_otu_table.py (input otu_table_metadata.biom and metadata file [step 1] to choose the category to split .biom file)

🡪 otu_split_metadata.biom

12. split_otu_table_by_taxonomy (input otu_table_metadata.biom to split specifically by taxonomic level. Adding –n 3 will split by phylum 🡪 output otu_table_Metazoans.biom

13. print_biom_table_summary.py (input otu_table_Metazoans.biom, generates statistics in command window for copy to text file)

14. convert_biome.py (input any biom file [steps 9,10,11,12], will convert files into tab delimitated file read by excel) 🡪 otu_table.txt

15. align_seqs.py (input rep_set.fna [step 5] to use PYNAST method to align the representative sequences) 🡪 rep_set_aligned.fasta

16. filter_aignment.py (input rep_set_aligned.fasta file to filter alignment) 🡪 rep_set_aligned_pfiltered.fasta

17. make_phylogeny.py (input rep_set_aligned_pfiltered.fasta to make tree file needed to run some alpha/beta diversity analyses)

🡪 rep_phylo.tre

18. summarize_taxa_through_plots.py (input otu_table_Metazoans.biom [step 12] and metadata file [step 1] to generate plots based on a category specified in the metadata file. Add –c and name of category to specify separation by that category [i.e. mesh size, location]

🡪 per_study_otu_tables.biom
