# Supplementary material for: Metabarcoding Is Powerful yet Still Blind: A Comparative Analysis of Morphological and Molecular Surveys of Seagrass Communities
Source: PLoS One. 2015 Feb 10;10(2):e0117562. doi: 10.1371/journal.pone.0117562 (PMC4323199; doi:10.1371/journal.pone.0117562)
Supplement: S1 Table — (DOCX) [file pone.0117562.s013.docx]

**S1 Table**

|  | **Gene** | |
| --- | --- | --- |
|  | **COI** | **18S** |
| No. of samples | 105 | 15 |
| No. of raw reads | 622,468 | 190,509 |
| Total sequences after filtering | 412,838 | 153,463 |
| Total sequences after chimera/singleton removal | 411,086 | 150,667 |
| Filtered average sequence length (bp) | 743 | 626 |
| Final OTU count for taxa assignment | 13,492 | 1,316 |
| Taxonomy assigned MOTUs | 944 | 1,268 |
| Taxonomy unassigned MOTUs | 12,548 | 48 |
