# Supplementary material for: Metabarcoding Is Powerful yet Still Blind: A Comparative Analysis of Morphological and Molecular Surveys of Seagrass Communities
Source: PLoS One. 2015 Feb 10;10(2):e0117562. doi: 10.1371/journal.pone.0117562 (PMC4323199; doi:10.1371/journal.pone.0117562)
Supplement: S3 Table — (DOCX) [file pone.0117562.s015.docx]

**S3 Table**

| **One-way 18S** | | | |
| --- | --- | --- | --- |
| *Meadow* | *Core* | | *Mesh* |
| R = 0.301, *p* = 0.013 | R = -0.124, *p* = 0.892 | | R = 0.257, *p* = 0.0142 |
|  | | | |
| **One-way COI** | | | |
| *Meadow* | *Core* | | *Mesh* |
| R = 0.766, *p* = 0.001 | R = -0.018, *p* = 0.945 | | R = 0.07, *p* = 0.001 |
|  | | | |
| **Two-way 18S** | | | |
| *Factor: Core* | | *Factor: Mesh* | |
| R = -0.625, *p* = 0.997 | | R = 0.352, *p* = 0.785 | |
|  | | | |
| **Two-way COI** | | | |
| *Factor: Core* | | *Factor: Mesh* | |
| R = 0.029, *p* = 0.185 | | R = 0.132, *p* = 0.010 | |
