# Supplementary material for: Metabarcoding Is Powerful yet Still Blind: A Comparative Analysis of Morphological and Molecular Surveys of Seagrass Communities
Source: PLoS One. 2015 Feb 10;10(2):e0117562. doi: 10.1371/journal.pone.0117562 (PMC4323199; doi:10.1371/journal.pone.0117562)
Supplement: S4 Table — SMG = Sainte Marguerite. (DOCX) [file pone.0117562.s016.docx]

**S4** **Table**

| **COI Meadow** | SMG_2010 | Arradon | Saint Malo | Roscanvel | Ile Callot | L’Arcouest | SMG_2011 |
| --- | --- | --- | --- | --- | --- | --- | --- |
| SMG_2010 |  | 0.0002 | 0.0001 | 0.0001 | 0.0001 | 0.0001 | 0.0001 |
| Arradon | 0.0002 |  | 0.0001 | 0.0001 | 0.0001 | 0.0001 | 0.0001 |
| Saint Malo | 0.0001 | 0.0001 |  | 0.0001 | 0.0001 | 0.0001 | 0.0001 |
| Roscanvel | 0.0001 | 0.0001 | 0.0001 |  | 0.0001 | 0.0001 | 0.0001 |
| Ile Callot | 0.0001 | 0.0001 | 0.0001 | 0.0001 |  | 0.0001 | 0.0001 |
| L’Arcouest | 0.0001 | 0.0001 | 0.0001 | 0.0001 | 0.0001 |  | 0.0001 |
| SMG_2011 | 0.0001 | 0.0001 | 0.0001 | 0.0001 | 0.0001 | 0.0001 |  |
|  | | | | | | | |
| **COI Mesh size** | | 0.5mm | | 1.0mm | | 2.0mm | |
| 0.5mm | |  | | 0.075 | | 0.000 | |
| 1.0mm | | 0.075 | |  | | 0.157 | |
| 2.0mm | | 0.000 | | 0.157 | |  | |
