# Supplementary material for: Metabarcoding Is Powerful yet Still Blind: A Comparative Analysis of Morphological and Molecular Surveys of Seagrass Communities
Source: PLoS One. 2015 Feb 10;10(2):e0117562. doi: 10.1371/journal.pone.0117562 (PMC4323199; doi:10.1371/journal.pone.0117562)
Supplement: S6 Table — (DOCX) [file pone.0117562.s018.docx]

**S6 Table**

| **18S Meadow** | | Sainte Marguerite | | Arradon | |
| --- | --- | --- | --- | --- | --- |
| Sainte Marguerite | |  | | 0.004 | |
| Arradon | | 0.004 | |  | |
|  | | | | | |
| **18S Mesh size** | 0.5mm | | 1.0mm | | 2.0mm |
| 0.5mm |  | | 0.084 | | 0.008 |
| 1.0mm | 0.084 | |  | | 0.161 |
| 2.0mm | 0.008 | | 0.161 | |  |
