# Supplementary material for: Metabarcoding Is Powerful yet Still Blind: A Comparative Analysis of Morphological and Molecular Surveys of Seagrass Communities
Source: PLoS One. 2015 Feb 10;10(2):e0117562. doi: 10.1371/journal.pone.0117562 (PMC4323199; doi:10.1371/journal.pone.0117562)
Supplement: S8 Table — Results with unassigned COI MOTUs included and excluded are reported (DOCX) [file pone.0117562.s020.docx]

**S8 Table**

| **Morphology vs. Molecular (COI)** | |
| --- | --- |
| Unassigned COI MOTUs included | R = 0.495, *p* = 0.177 |
| Unassigned COI MOTUs excluded | R = 0.641, *p* = 0.327 |
