# Supplementary material for: Metabarcoding Is Powerful yet Still Blind: A Comparative Analysis of Morphological and Molecular Surveys of Seagrass Communities
Source: PLoS One. 2015 Feb 10;10(2):e0117562. doi: 10.1371/journal.pone.0117562 (PMC4323199; doi:10.1371/journal.pone.0117562)
Supplement: S9 Table — (DOCX) [file pone.0117562.s021.docx]

**S9 Table**

| **One-way Morphology** | | |
| --- | --- | --- |
| *Meadow* | | *Core* |
| R = 0.924, *p = 0.001* | | R = -0.05, *p = 0.995* |
| **Two-way Morphology** | | |
| *Factor: Meadow* | *Factor: Core* | |
| R = 0.890, *p* = 0.001 | R = -0.196, *p* = 0.995 | |
