# Supplementary material for: Metabarcoding Is Powerful yet Still Blind: A Comparative Analysis of Morphological and Molecular Surveys of Seagrass Communities
Source: PLoS One. 2015 Feb 10;10(2):e0117562. doi: 10.1371/journal.pone.0117562 (PMC4323199; doi:10.1371/journal.pone.0117562)
Supplement: S10 Table — SMG = Sainte Marguerite. (DOCX) [file pone.0117562.s022.docx]

**S10 Table**

| **Morph Meadow** | Arradon | Saint Malo | Roscanvel | Ile Callot | L’Arcouest | SMG |
| --- | --- | --- | --- | --- | --- | --- |
| Arradon |  | 0.0003 | 0.0002 | 0.0001 | 0.0001 | 0.0002 |
| Saint Malo | 0.0003 |  | 0.0002 | 0.0002 | 0.0001 | 0.0002 |
| Roscanvel | 0.0002 | 0.0002 |  | 0.0001 | 0.0001 | 0.0001 |
| Ile Callot | 0.0001 | 0.0002 | 0.0001 |  | 0.0001 | 0.0001 |
| L’Arcouest | 0.0001 | 0.0001 | 0.0001 | 0.0001 |  | 0.0001 |
| SMG | 0.0002 | 0.0002 | 0.0001 | 0.0001 | 0.0001 |  |
